# Beyond bronchial thermoplasty – where to now?

**DOI:** 10.1016/j.eclinm.2024.103017

**Published:** 2024-12-21

**Authors:** Peter B. Noble, David Langton, Chuan T. Foo, Bruce R. Thompson, Alvenia Cairncross, Michael J. Hackmann, Francis Thien, Graham M. Donovan

**Affiliations:** a School of Human Sciences, The University of Western Australia, WA, Australia; b Faculty of Medicine, Nursing and Health Sciences, Monash University, Melbourne, VIC, Australia; c Department of Thoracic Medicine, Peninsula Health, Frankston, VIC, Australia; d Department of Respiratory Medicine, Eastern Health, Melbourne, VIC, Australia; e School of Health Sciences, University of Melbourne, VIC, Australia; f Department of Pulmonary Physiology and Sleep Medicine, Sir Charles Gairdner Hospital, WA, Australia; g Department of Mathematics, University of Auckland, Auckland, New Zealand

**Keywords:** Airway smooth muscle, Bronchial thermoplasty, Asthma, Structure-guided therapy

## Abstract

With the impending ‘retirement’ of bronchial thermoplasty (BT) for the treatment of patients with asthma, there is much to learn from this real-world experiment that will help us develop more effective future therapies with the same primary target i.e., airway smooth muscle (ASM) remodelling. This viewpoint discusses initial controversy surrounding BT (lack of an effect on forced expiratory volume in 1 s), its underutilisation, and importantly how non-standard diagnostics successfully demonstrated therapeutic response which escaped traditional lung function metrics. It is anticipated that the next iteration of BT (likely in a drug form) will have an overall greater effect on the health care system by virtue of evoking ASM remodelling as a treatable trait, and after appropriately drawing on lessons learned from the ∼fifteen-year BT saga.

## Introduction

Almost 15 years have passed since direct therapeutic targeting of airway smooth muscle (ASM) remodelling was made possible through the approval of bronchial thermoplasty (BT) in the management of severe and uncontrolled asthma.^1^ From one perspective, BT has been a success, producing persisting (permanent) improvements in symptom control and exacerbation rates that are observed even 10 years post-treatment.^2^ On the other hand, BT utilisation has been very low (as few as ∼500 patients/year from 2007 to 2019),^3^ and in this regard has failed in the healthcare system. It is reasonable to assume this unfavourable commercialisation environment has led to the recent announcement by the manufacturer to discontinue sales of BT products (S1). There are nonetheless important lessons to be drawn from the BT experiment about how we assess therapeutic response that will allow us to prudently plan for interventions targeting ASM remodelling as a treatable trait beyond BT.

In brief, for appropriate context, BT can be described as a specialist non-pharmacological therapy for asthma. Under bronchoscopic guidance, a BT catheter is inserted into the airway lumen where a basket coil tip makes contact with the epithelial surface, emitting radiofrequency energy that heats airway tissue to 65 °C. Airways 2–10 mm in diameter are typically treated by BT, across three separate procedural sessions, each covering one out of the following regions of the lung; right lower lobe, left lower lobe, and both upper lobes. The right middle lobe is traditionally left untreated due to concerns of right middle lobe syndrome, although recent data suggests that treatment of the middle lobe may be safe.^4^ For respiratory medicine, BT was a relatively bold approach, initially inducing further tissue disruption to already inflamed and structurally abnormal airways. Nevertheless, the motivation behind BT was logical, representing the only proven treatment to reduce ASM remodelling.

## Motivation behind bronchial thermoplasty: direct targeting of airway smooth muscle

In patients with asthma the ASM layer is thickened (‘remodelled’),^5^ contributing mechanically to a myriad of clinical and pathophysiological manifestations, most notably bronchospasm with reversible airflow obstruction and airway hyperresponsiveness.^6^ The origin of ASM remodelling in asthma (how ASM thickness increased) is in dispute; most favour a mechanism downstream to inflammation,^7^ but there are other possible explanations too, including an abnormality produced by some developmental disorder.^8^ Heat-induced ablation of ASM was really a shrewd strategy to correct a structural defect where the underlying cause was not entirely clear, and one not reliant on resolution of underlying inflammatory mediators. That is, when using BT, it didn't matter why the ASM was thickened in the first place, as the primary effector tissue in asthma was susceptible to excess heat and could be directly atrophied in this manner. Using ASM area as a primary endpoint, BT most certainly works, producing a consistent decrease in the area of ASM in biopsy,^9^ with no evidence of regrowth when assessed >2.5 years post-treatment.^10^

## The clinical conundrum; how does bronchial thermoplasty work?

Availability of alternative therapies, particularly revolutionary monoclonal antibodies, is part of the reason why physicians have not turned regularly to BT, despite evidence from a systematic review and meta-analysis showing that BT is non-inferior to biologics.^11^ There still remain a large patient pool of non-atopic, non-eosinophilic patients with asthma who do not qualify for, or do not achieve sufficient disease control with biologics. A surgical solution for what is considered largely an inflammatory disorder also challenges conventions. BT is comparatively invasive, requiring bronchoscopy, anaesthesia, and prophylactic oral corticosteroids to minimise post-procedural side-effects.

A major reason for physician reluctance to prescribe BT is the lack of a clear understanding of how BT actually improves asthma control. From inception, BT was controversial, with many researchers and clinicians believing that consistent improvements in patient-reported outcomes (symptoms and healthcare usage) were driven by placebo effects, given the lack of consistent reported changes in traditional functional metrics (e.g., forced expiratory volume in 1 s, FEV_1_).^12^ Even after a sham-controlled trial,^13^ the perception that BT response was confounded by placebo has been hard to shake. Of course (many would say) BT treats a limited number of relatively large airways which would never affect lung function! To be fair, when mathematical modelling studies on BT were designed there was not a great deal of optimism that therapy localised to the large airways would result in a meaningful change in lung function; somewhat surprisingly a beneficial effect was predicted by the simulations, with a caveat that simple spirometry would likely be insensitive to any response,^14^ discussed further below. The explanation is related to interdependence between proximal and distal airways and with surrounding lung parenchyma that allows pathologies in one region of the lung to affect another,^15^ and so therapy directed at the large airways impacts small airway function.^14^ Put simply, treatment of large airways with BT facilitates more advantageous flow profiles in the lung periphery.

The view that BT effects on lung function go undetected (or are not there at all) is at best outdated, and at worst, just wrong. Patients receiving BT have dilation of large airways on computed tomography (CT)^16^ and reduced airway resistance assessed using plethysmography.^17^ Both CT^18^ and plethysmography^19^ have been used to demonstrate reduced gas trapping after BT. Ultimately BT seems to improve the efficiency of breathing by driving a more homogenous ventilation of the lung. BT lowers ventilation heterogeneity when assessed by polarised xenon-magnetic resolution imaging,^20^ functional respiratory imaging CT^21^ and lung clearance index by multiple breath nitrogen wash-out.^22^ Notably, ventilation heterogeneity is a determinant of poor asthma control^23^ and, independent of inflammation, airway hyperresponsiveness.^24^

## What have we then learnt from bronchial thermoplasty?

Taken together, these results strongly suggest that BT induces non-placebo physiological changes, and this leads to improved patient outcomes without being readily reflected in traditional measures of lung function. This is how we, as a community, missed BT's effects and mechanism of action in its early days. While not necessarily a paean to BT, since many other factors go into treatment selection, the sensitivity or insensitivity of various lung function measures to interventions like BT has important implications going forward on how we should be assessing efficacy of future treatments.

The lung is a complex and difficult thing; the airways do not function in isolation, but instead as an interrelated and interconnected complex system, exhibiting sometimes counterintuitive phenomena.^15^ That we on occasions have difficulty understanding and measuring this behaviour does not make it less so. In the end we should care most about health outcomes, even if they are inconveniently contaminated as statistical measures by patient perceptions. Spirometry is certainly the current clinical gold standard, for good reason in many cases, but it is not perfect. If a treatment is improving patient outcomes, but not spirometry, we need to ask ourselves what our functional measurements are overlooking, rather than assuming there are simply no underlying changes.

It would be tempting to simply recommend, as an alternative to spirometry, one of the metrics that do consistently reflect BT's physiological changes. However, this could be misleading, because these tests also have their own limitations and give only a partial indication of lung function. Instead, we must remember that different metrics show us different aspects of a difficult-to-measure system and may each have value in different contexts. As much as we might like to have one gold standard, there is no single source of truth when it comes to lung function, and pretending otherwise can overlook changes that improve patient health. Basic science and theoretical models can contribute, at the very least by helping us know where to look.

## Beyond bronchial thermoplasty

If designing a therapy from scratch that targets ASM remodelling, the key advancements would be to treat both large and small airways (only the former being accessible by BT), not simply blindly treating patients who may or may not have ASM remodelling, and ideally avoiding surgical intervention altogether. All can be achieved through use of a pharmacological therapy targeting ASM remodelling, which should be especially efficacious if administered to patients with the appropriate structural phenotype. Structural ASM remodelling phenotypes have been documented,^25^ where some patients have wide-spread ASM remodelling, and others little to no ASM remodelling. Again, the answer comes down to the capacity to effectively measure, this time the extent and magnitude of ASM remodelling. Biopsy has been a temporary solution, albeit a non-representative (limited to large airways) minuscule sample that is not feasible for extended use in patients with asthma. Translation of bronchoscopic-guided polarisation-sensitive optical coherence tomography, a laser based optical imaging method, is in the pipeline and able to accurately quantify ASM remodelling in a non-injurious manner,^26^, ^27^, ^28^ in large and small airways, with the capacity to cover a sampling area that is orders of magnitude greater than biopsy.

Key remaining questions are–what pharmaceuticals can resolve ASM remodelling and how will patients be managed? A series of clinical studies and drug trials are anticipated now that measurement of ASM remodelling throughout the bronchial tree appears feasible in patients. Preliminary data on an effect of mepolizumab in reducing ASM area in biopsy was presented at the European Respiratory Society congress last year^29^ and we note good pre-clinical data demonstrating that azithromycin also reduces ASM thickness.^30^^,^^31^ Eventually the treatment algorithm may include diagnostic assessment of ASM remodelling and/or similarly sensitive lung function tests,^32^ identifying patients who should be treated with an ASM remodelling targeting pharmaceutical and indeed who is likely to respond. As an exemplar, a recent study in chronic obstructive pulmonary disease showed that patients with high ASM content in biopsy had a clinically meaningful improvement in FEV_1_ after corticosteroid compared to those with low ASM,^33^ demonstrating the utility of assessing ASM remodelling in patients before deciding on a treatment approach.

## Conclusions

So as BT begins its farewell (1–2 years depending on how long the catheter stock lasts), it can be said that BT has not only improved the symptom control and exacerbation rates of the patients actually treated with BT but has given rise to a line of investigation that will help evolve the respiratory health care model. BT should and will not be the last therapy that targets ASM remodelling, although successors will likely materialise differently to the first iteration, both in terms of application and assessment of therapeutic response. With the benefit of experience and thus a more informed interpretation of outcomes produced by therapies targeting ASM remodelling as a treatable trait, the beyond-BT era can draw from these lessons and have an overall greater impact on reducing the burden of asthma.

## Contributors

Authors collectively conceived the subject of the viewpoint and outlined broad organisation. PN and GD drafted the manuscript. DL, CF, BT, AC, MH and FT provided editorial changes and feedback. All authors approved the final version of the manuscript.

## Data sharing statement

Not applicable to this viewpoint article which did not present any original data.

## Declaration of interests

The authors declare no conflict of interest.
