# Supplementary material for: Beyond bronchial thermoplasty – where to now?
Source: eClinicalMedicine. 2024 Dec 21;79:103017. doi: 10.1016/j.eclinm.2024.103017 (PMC11731591; doi:10.1016/j.eclinm.2024.103017)
Supplement: Supplement [file mmc1.pdf]

Notice of Product Discontinuation

Dear Valued Customer:
After careful consideration, Boston Scientific has decided to discontinue sales of the Alair™ Bronchial Thermoplasty System globally over the coming years. This is due to a changing regulatory environment and current market conditions that have made it a challenge to sustain this technology.

We want you to know this is not a decision that has been made lightly, as Boston Scientific has championed bronchial thermoplasty for more than 12 years. We remain committed to innovating in the pulmonary space to advance care for patients around the world

For your reference, key discontinuation dates in all global regions outside of EMEA* are:

- Alair™ Bronchial Thermoplasty Catheters and Accessory Kits will be sold through **December 31, 2024**
- Alair™ Bronchial Thermoplasty Controllers will be sold through **December 31, 2022**
- Alair™ Bronchial Thermoplasty Controllers will be serviced through **December 31, 2025****

The items and ordering numbers directly associated with this discontinuation are listed below:

Discontinued Product UPN	Discontinued Product Description	Discontinuation Date
**M005ATS20000**	Alair Bronchial Thermoplasty Controller	December 31, 2022 (sold), December 31, 2025 (service)*
**M005ATS25010**	Alair Bronchial Thermoplasty Catheter – North America***	December 31, 2024
**M005ATS25020**	Alair Bronchial Thermoplasty Catheter - Global	December 31, 2024
**M005ATS201xx**	Alair Accessory Kit- all models	December 31, 2024

* For Customers in Europe Middle East and Africa (EMEA) please contact your local representative as the timelines are different
**December 31, 2025 is the latest date that Alair Bronchial Thermoplasty Controllers will be serviced. Actual service termination dates are dictated by the service agreement with individual hospitals.
*** M005ATS25010 also applies to India, China and Japan outside of North America

If you have any questions, please contact your Boston Scientific sales representative.

Sincerely,
Noah Webster
Principal Global Product Manager

CAUTION: U.S. Federal law restricts these devices to sale by or on the order of a physician. Indications, contraindications, warnings, and instructions for use can be found in the product labelling supplied with each device or at www.IFU-BSCI.com.
2022 Copyright © Boston Scientific Corporation or its affiliates. All rights reserved. ENDO-1366207-AA
